# Supplementary material for: Plasma and urine metabolomic analyses in aortic valve stenosis reveal shared and biofluid-specific changes in metabolite levels
Source: PLoS One. 2020 Nov 25;15(11):e0242019. doi: 10.1371/journal.pone.0242019 (PMC7688110; doi:10.1371/journal.pone.0242019)
Supplement: S1 Table — Data are means ± SEM. (DOCX) [file pone.0242019.s001.docx]

**S1 Table:** Clinical and biochemical features in males and females selected for presence or absence of aortic valve stenosis. Data are means ± SEM.

|  | Females (38) | | Males (54) | |
| --- | --- | --- | --- | --- |
|  | Controls (19) | Cases (19) | Controls (27) | Cases (27) |
| Age | 61.8 ± 2.7 | 61.4 ± 2.6 | 57.3 ± 2.5 | 57.0 ± 2.6 |
| Body weight (Kg) | 75.2 ± 3.3 | 73.4 ± 3.0 | 88.6 ± 3.0 | 87.4 ± 3.0 |
| Body mass index (Kg/m^2^) | 31.8 ± 1.3 | 31.7 ± 1.2 | 30.0 ± 0.9 | 30.1 ± 0.9 |
| Plasma glucose (mg/dL) | 122.3 ± 12.4 (17) | 116.3 ± 23.8 (4) | 102.6 ± 5.0 (19) | 111.0 ± 5.3 (9) |
| Triglycerides (mg/dL) | 207.4 ± 22.3 (19) | 170.5 ± 21.1 (16) | 167.6 ± 14.4 (23) | 198.0 ± 14.4 (22) |
| HDL cholesterol (mg/dL) | 44.4 ± 3.2 (19) | 47.8 ± 3.2 (16) | 36.3 ± 1.9 (24) | 34.6 ± 2.2 (23) |
| LDL cholesterol (mg/dL) | 126.9 ± 11.3 (19) | 109.4 ± 8.0 (15) | 114.3 ± 6.4 (23) | 109.4 ± 8.0 (23) |
| Total cholesterol (mg/dL) | 199.7 ± 14.6 (19) | 184.8 ± 8.3 (16) | 183.5 ± 8.0 (24) | 185.7 ± 9.1 (23) |
| Diagnosed diabetic (%) | 5 (26.3%) | 5 (26.3%) | 2 (7.4%) | 6 (22.2%) |
| Diagnosed hypertensive (%) | 15 (78.9%) | 14 (73.7%) | 12 (44.4%) | 22 (81.5%) |
| Diagnosed hyperlipidemic (%) | 10 (52.6%) | 13 (68.4%) | 2 (7.4%) | 15 (55.6%) |
| Family history diabetes (%) | 12 (63.2%) | 9 (47.4%) | 17 (63.0%) | 14 (51.9%) |
| Family history hypertension (%) | 15 (78.9%) | 15 (78.9%) | 19 (70.4%) | 19 (70.4%) |
| Family history hyperlipidemia (%) | 11 (57.9%) | 9 (47.4%) | 5 (18.5%) | 12 (44.4%) |
