# Supplementary figures and images for: Plasma and urine metabolomic analyses in aortic valve stenosis reveal shared and biofluid-specific changes in metabolite levels
Source: PLoS One. 2020 Nov 25;15(11):e0242019. doi: 10.1371/journal.pone.0242019 (PMC7688110; doi:10.1371/journal.pone.0242019)

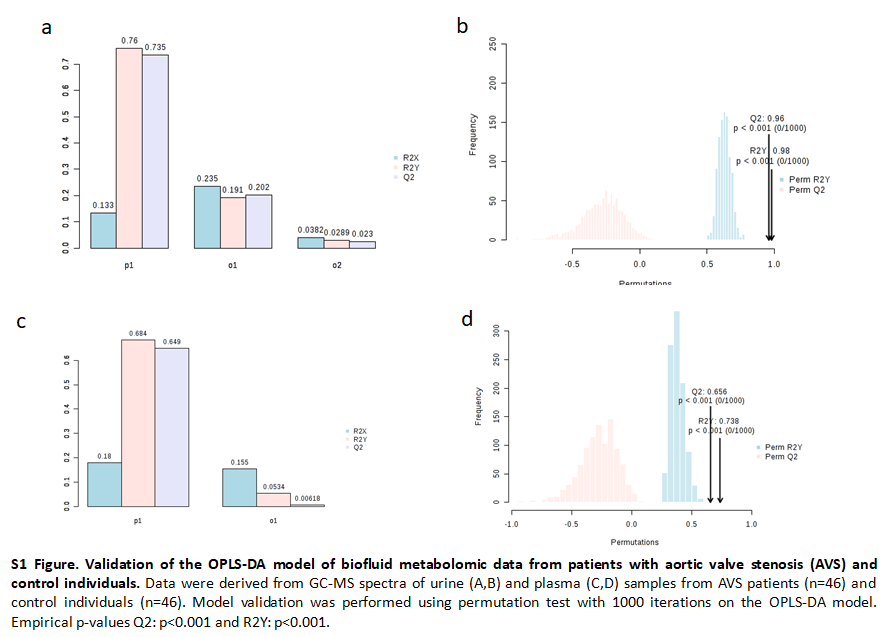

Supplement: S1 Fig — Data were derived from GC-MS spectra of urine (a, b) and plasma (c, d) samples from AVS patients (n = 46) and control individuals (n = 46). Model validation was performed using permutation test with 1000 iterations on the OPLS-DA model. Empirical p-values Q2: p<0.001 and R2Y: p<0.001. (TIF) [file pone.0242019.s007.tif]

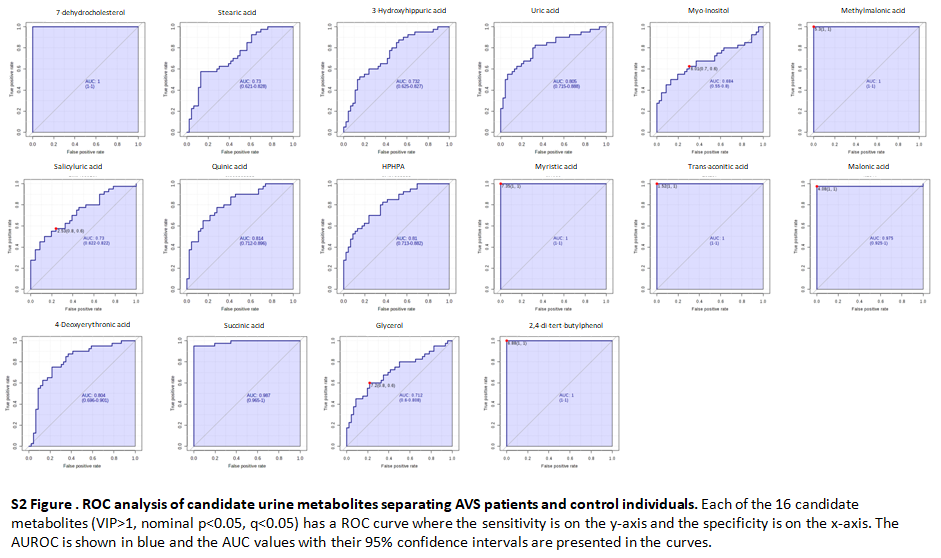

Supplement: S2 Fig — Each of the 16 candidate metabolites (VIP>1, nominal p<0.05, q<0.05) has a ROC curve where the sensitivity is on the y-axis and the specificity is on the x-axis. The AUROC is shown in blue and the AUC values with their 95% confidence intervals are presented in the curves. (TIF) [file pone.0242019.s008.tif]

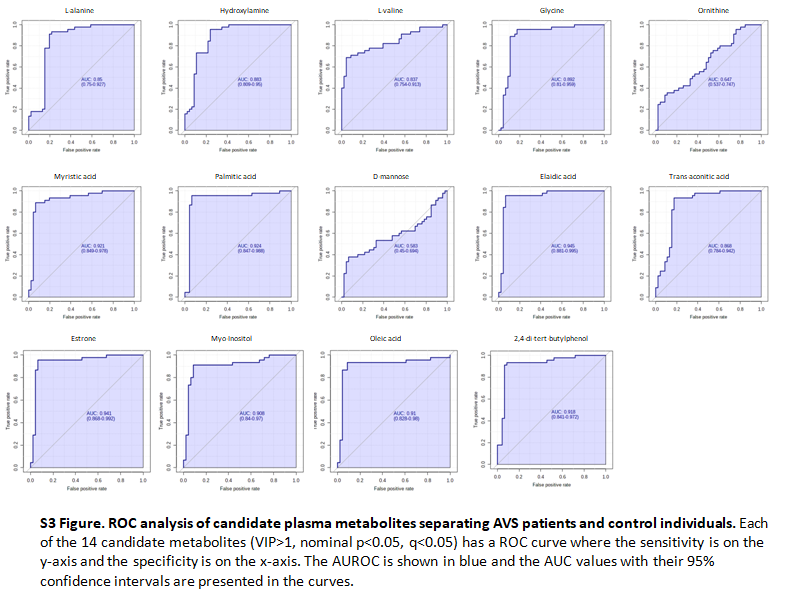

Supplement: S3 Fig — Each of the 14 candidate metabolites (VIP>1, nominal p<0.05, q<0.05) has a ROC curve where the sensitivity is on the y-axis and the specificity is on the x-axis. The AUROC is shown in blue and the AUC values with their 95% confidence intervals are presented in the curves. (TIF) [file pone.0242019.s009.tif]

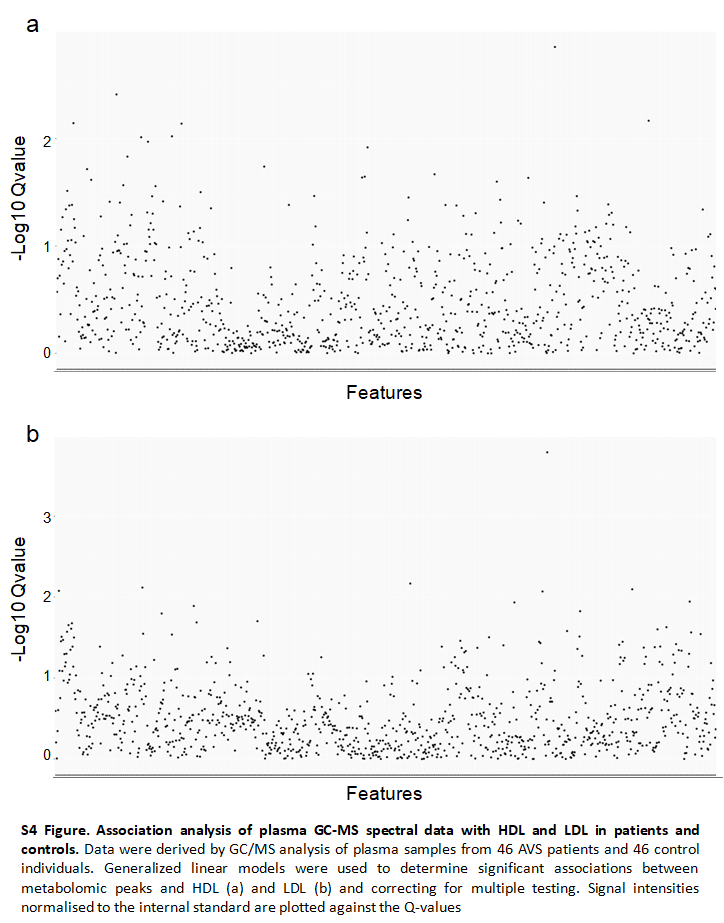

Supplement: S4 Fig — Data were derived by GC-MS analysis of plasma samples from 46 AVS patients and 46 control individuals. Generalized linear models were used to determine significant associations between metabolomic peaks and HDL (a) and LDL (b) and correcting for multiple testing. Signal intensities normalized to the internal standard are plotted against the Q-values. (TIF) [file pone.0242019.s010.tif]
