# Supplementary material for: Plasma and urine metabolomic analyses in aortic valve stenosis reveal shared and biofluid-specific changes in metabolite levels
Source: PLoS One. 2020 Nov 25;15(11):e0242019. doi: 10.1371/journal.pone.0242019 (PMC7688110; doi:10.1371/journal.pone.0242019)
Supplement: Attachment — Submitted filename: PONE-D-20-24273_R2_Response to Reviewers.docx [file pone.0242019.s011.docx]

**Comments to the Author**
1. If the authors have adequately addressed your comments raised in a previous round of review and you feel that this manuscript is now acceptable for publication, you may indicate that here to bypass the “Comments to the Author” section, enter your conflict of interest statement in the “Confidential to Editor” section, and submit your "Accept" recommendation.

Reviewer #1: All comments have been addressed

Reviewer #2: All comments have been addressed

Reviewer #3: All comments have been addressed

2. Is the manuscript technically sound, and do the data support the conclusions?
Reviewer #1: Yes

Reviewer #2: Yes

Reviewer #3: Yes

3. Has the statistical analysis been performed appropriately and rigorously?

Reviewer #1: Yes

Reviewer #2: Yes

Reviewer #3: Yes

4. Have the authors made all data underlying the findings in their manuscript fully available?
Reviewer #1: Yes

Reviewer #2: Yes

Reviewer #3: Yes

5. Is the manuscript presented in an intelligible fashion and written in standard English?
Reviewer #1: Yes

Reviewer #2: Yes

Reviewer #3: Yes

6. Review Comments to the Author
Reviewer #1: (No Response)

Reviewer #2: The authors have answered all comments, but one: the addition of a correction for urine dilution is feasible and would have been highly relevant to the study.

Response: We agree with the Reviewer that urine dilution is an important criterion when analysing urine metabolomics. As a rule, we also have to deal with correction for technical deviation between samples and runs using the internal standard in order to consistently normalise the urine and plasma datasets with exactly the same method.

As requested by the Reviewer, we have recalculated the statistics using urine data normalised to creatinine instead of the internal standard. We have mentioned this in the methods section.

The results from association studies between urine metabolites and AVS are very concordant in both cases of data normalisation. We now report in a new table in the revised version of the manuscript (Supplementary Table 4) results from the association between AVS and urine metabolites normalised to creatinine before adjustment for age, sex, body mass index, hyperlipidemia and diabetes.

Also, we have changed the format of Table 2 to show in parallel association results, following adjustment for age, sex, body mass index, hyperlipidemia and diabetes, based on urine metabolomic data independently normalised to the internal standard and to creatinine. This provides the reader with elements to directly compare association data using these two methods, and to be able to visualise consistency in the outputs. Essentially, almost all associations identified following normalisation to the internal standard are replicated when the data are normalised to creatinine, suggesting higher stringency upon data normalisation to the internal standard.

Details of these new findings are given in the results section, pages 11 to 14.

A reference (Fiehn O. Metabolomics by Gas Chromatography-Mass Spectrometry: Combined Targeted and Untargeted Profiling. Curr Protoc Mol Biol. 2016;114:30.4.1-.4.2) was added.

Minor comments:
*Lines 264-268: the two sentences appear contradictory. Please edit the first one to clarify that the series of metabolites are consistent within (not across) fluids.

Response: The reviewer is correct. The sentences have been changed (lines 288-293 of the revised manuscript).

*Figures 5 and 6:
-What is the y-axis legend ? what units? what numbers (impossible to read)?
-Display the controls on the left and cases on the right to help readers

Response: Figures 5 and 6 have been redrawn as requested by the Reviewer. Font has been increased and data from controls and cases are shown on the left and right, respectively, for each panel. Details of the unit of the Y-axis are given in the legends to the figures.

*Figure 7:
-identify starch metabolism

Response: As requested, starch metabolism, which was omitted on the original version of Figure 7, was added in the revised version of this Figure. The text on page 15 was amended. We apologise for this mistake.

-display backtransformed P-values on the y-axis (and color legend).

Response: As requested, the revised version of Figure 7 displays backtransformed P-values on the y-axis.

Reviewer #3: Thank you for taking the time to make the requested amendments. I have nothing further to add.

7. PLOS authors have the option to publish the peer review history of their article ([what does this mean?](https://journals.plos.org/plosone/s/editorial-and-peer-review-process#loc-peer-review-history)). **Do you want your identity to be public for this peer review?** For information about this choice, including consent withdrawal, please see our [Privacy Policy](https://www.plos.org/privacy-policy).

Reviewer #1: No

Reviewer #2: No

Reviewer #3: No
